# 

**Published:** 1897-11

**Authors:** 


### Obituary.

### REPORT OF THE COMMITTEE ON NECROLOGY OF THE NATIONAL ASSOCIATION OF DENTAL FACULTIES, AT OLD POINT COMFORT.

### PROFESSOR FRANK ABBOT, OF NEW YORK N. Y.

*Whereas*, Death has removed from our ranks Prof. Frank Abbott, of New York; and whereas, on account of his social qualities, his genial companionship, and his ability as a practitioner and teacher of dentistry, we realize the great loss to the profession in his death; therefore be it

*Resolved*, That we tender to his family the sincere sympathy of this association, and request that these resolutions be spread upon the minutes of the association; and be it further

*Resolved*, That a copy of these resolutions be sent to the several dental journals of this country for publication.

### DR. FRANCIS PEABODY, OF LOUISVILLE, KY.

*Whereas*, Death has taken from among us Dr. Francis Peabody, of Louisville, Ky.; and whereas, we feel that in his death the profession has sustained the loss of an able practitioner and teacher; therefore be it

*Resolved*, That we tender to his bereaved family our heartfelt sympathy, and that we cause these resolutions to be entered upon the minutes of this association; and be it further

*Resolved*, That a copy of these resolutions be sent to the dental journals for publication.

### Bibliographical.

THE AMERICAN TEXT BOOK OF PROSTHETIC DENTISTRY. By eminent American authorities. Editor : Charles J. Essig, M. D., D. D. S., Professor of Mechanical Dentistry and Metallurgy in the Dental Department of the University of of Pennsylvania. Publishers : Lea Brothers & Co., Philadelphia and New York, 1896.

### To Readers and Correspondents.

Communications intended for publication, and exchange journals and papers, should be addressed to the Editor of The American Journal of Dental Science, No. 845 N. Eutaw St. Baltimore, Md. All letters relating to the business of the Journal, or containing cash remittances, to be directed to Snowden & Cowman Mfg. Co., 9 W. Fayette Street, Baltimore, Md. Articles for publication should be received by the Editor at least two weeks previous to the first of the month on which the number in which it is designed for them to appear is issued.

☞ The American Journal of Dental Science will contain fifty pages of reading matter in each number, making a volume of about Six Hundred pages,

EXCLUSIVE OF ADVERTISEMENTS.

